# The cost of free health care for all Kenyans: assessing the financial sustainability of contributory and non-contributory financing mechanisms

**DOI:** 10.1186/s12939-017-0535-9

**Published:** 2017-02-27

**Authors:** Vincent Okungu, Jane Chuma, Di McIntyre

**Affiliations:** 1 Institute of Healthcare Management, Strathmore University, Nairobi, Kenya; 2 KEMRI-Wellcome Trust Research Programme, Nairobi, Kenya; 3 Health Economics Unit, University of Cape Town, Cape Town, South Africa

**Keywords:** Universal health coverage, Informal sector, Contributory, Non-contributory

## Abstract

**Background:**

The need to provide quality and equitable health services and protect populations from impoverishing health care costs has pushed universal health coverage (UHC) to the top of global health policy agenda. In many developing countries where the majority of the population works in the informal sector, there are critical debates over the best financing mechanisms to progress towards UHC. In Kenya, government health policy has prioritized contributory financing strategy (social health insurance) as the main financing mechanism for UHC. However, there are currently no studies that have assessed the cost of either social health insurance (SHI) as the contributory approach or an alternative financing mechanism involving non-contributory (general tax funding) approaches to UHC in Kenya. The aim of this study was to critically assess the financial requirements of both contributory and non-contributory mechanisms to financing UHC in Kenya in the context of large informal sector populations.

**Methods:**

SimIns Basic® model, Version 2.1, 2008 (WHO/GTZ), was used to assess the feasibility of UHC in Kenya and provide estimates of financial resource needs for UHC over a 17-year period (2013–2030). Data sources included review of national and international literature on inflation, demography, macro-economy, health insurance, health services unit costs and utilization rates. The data were triangulated across geographic regions for accuracy and integrity of the simulation. SimIns models for 10 years only so data from the final year of the model was used to project for another 7 years. The 17-year period was necessary because the Government of Kenya aims to achieve UHC by 2030.

**Results and conclusions:**

The results show that SHI is financially sustainable (Sustainability in this study is used to mean that expenditure does not outstrip revenue.) (revenues and expenditure match) within the first five years of implementation, but it becomes less sustainable with time. Modelling for a non-contributory scenario, on the other hand, showed greater sustainability both in the short- and long-term. The financial resource requirements for universal access to health care through general government revenue are compared with a contributory health insurance scheme approach. Although both funding options would require considerable government subsidies, given the magnitude of the informal sector in Kenya and their limited financial capacity, a tax-funded system would be less costly and more sustainable in the long-term than an insurance scheme approach. However, more innovative financing for health care as well as giving the health sector higher priority in government expenditure will be required to make the non-contributory financing mechanism more sustainable.

## Background

Universal health coverage (UHC) is a priority policy agenda worldwide and is one of the Sustainable Development Goals (SDGs). The SDG3, where UHC falls, is driven by the need for improved access to quality health services for all and protection of the population from catastrophic and impoverishing health care costs. Access to health care services should be equitable and sustainable, based on need and not ability to pay [1]. The design of health financing systems has important implications for UHC. Countries that finance their health systems predominantly through mandatory prepayments including contributory (social health insurance) and non-contributory mechanisms (general government revenues), make faster progress to UHC [2]. The World Health Organization (WHO) [1] regards mandatory prepayment as the most efficient and equitable financing system for UHC. Mandatory prepayment for services are preferred because they potentially generate high revenue, promote risk and income cross-subsidization and minimize financial barriers to access [3]. Because they tend to include the entire population or a large majority of it, mandatory prepayment systems address problems of adverse selection, are financially secure and benefit from economies of scale as well as contribute to the improvement of equity in the distribution of health resources [4, 5]. Moreover, they are domestic financing sources and this makes them more sustainable and predictable for the long term.

A critical point for countries that are reforming their health systems to progress to UHC is the need to consider total resource requirements over the long term to plan for the implementation and sustainable financing of UHC. The decision on the appropriate mix of financing mechanisms should be informed by evidence particularly on the feasibility of their revenue generating potential, over a period of time to allow for long-term planning and budgeting.

In Kenya, the importance of financial protection to UHC cannot be overemphasized. About 83% of Kenyans lack financial protection from health care costs, and about 1.5 million Kenyans are pushed into poverty each year as a result of paying for health care [6]. The Kenyan Government is committed to moving towards UHC and is currently developing a health financing strategy that will provide an overarching policy framework for health financing reforms. Ongoing policy discussions indicate that mandatory health insurance, where both formal and informal sector workers pay a premium into a national pool, will be the predominant health financing mechanism for Kenya. However, questions have recently been raised regarding the feasibility of a contributory approach in settings with large informal sector populations for various reasons including: the difficulty of determining incomes of informal sector workers; appropriate premium rates; how to enforce contributions and ensure that revenue collection mechanisms are administratively efficient. Besides, even if incomes were determinable in the informal sector, the premium contributions would be very onerous because of generally low-incomes in the sector. The experiences of Thailand and Ghana confirm that determining incomes and identifying the poor in the informal sector are problematic [7, 8]. Universal health coverage has major monetary implications for the government, firms and households which need to be considered over the long-term. It is important to consider all financing approaches for UHC to arrive at a strategy that has the lowest possible costs, equitably distributes the financing burden and ensures equity of access to services. Before Kenya disregards possibilities of funding the health system through a non-contribution model it is important to assess the resource potential of a tax funded system, as an alternative to the contributory approach. This paper models the resource requirements of the two financing approaches to UHC, and assesses their feasibility in the Kenyan context.

## Methods

### Simulation Insurance (SimIns) modelling

The feasibility of UHC in Kenya was modelled using SimIns Basic® (GIZ/WHO). SimIns models for a 10 year period, so the study first modelled from 2013 to 2023 and then used the 2023 data as second round input to extend the model to 2030. Details on how SimIns Basic works are provided in the User Guide [9]. As a consequence of expanding coverage under a contributory mechanism, health facilities will be expected to gradually meet most of their expenses from payments by the health insurance organization, meaning that there will be less funds going directly from general government revenue to support public sector facilities. In this context, SimIns Basic is able to demonstrate the trend in health insurance funding as it gradually replaces the flow of government funds into public sector health facilities. The aim of the simulation is therefore, to demonstrate separately, the feasibility of UHC through (i) social health insurance supplemented by government revenues and, (ii) funding from general government revenues supplemented by premium contributions from the formal sector (given that mandatory contributions by the formal sector already exist within Kenya).

### Scenarios for financing UHC

In the proposed contributory financing policy, all Kenyans including formal and informal sector workers, pensioners and the indigent are required to belong and contribute premiums into one pool. Within this pool, formal and informal sector workers contribute premiums on a regular basis whereas the government pays premiums for the indigent. Currently, those in the informal sector who have voluntarily joined the NHIF contribute a flat-rate of Kenya Shillings (KSh) 500 (about US$ 5.00) per household on a monthly basis for outpatient and inpatient services. Contributions from the formal sector are currently based on salary scales capped at a salary of KSh 100,000 (US$ 1,000) per month. The contributions from the formal sector on average represent about 2.4% of average gross pay. The financing scenarios for UHC are described in Table 1. The scenarios described are: (a) premium contributions as the main financing strategy and (b) general government revenues as the main financing strategy.

**Table 1:** Summary of two financing scenarios explored in the SimIns basic model

Scenario 1: Contributory system: Social health insurance (SHI) scheme This scenario mirrors the preferred government financing model for UHC in which all Kenyans are expected to contribute premiums to a scheme (SHI). This financing scenario retains most of the design features of the current public insurer, the National Hospital Insurance Fund (NHIF), which is the preferred government organization for UHC. Under Scenario 1, government funding plays a complementary role to premium contributions. With the majority of formal sector workers already covered by the NHIF, the design of a future scheme is targeted at gradually enrolling and retaining informal sector workers through a mix of strategies including wireless premium payments and devolved registration centers and an expanded benefits package. Some of these strategies are already in place. Formal and informal sector workers are expected to pay standardized premium rates but the government will pay contributions for the indigent. It is not clear how the indigent will be identified. All population groups are expected to be under a single national pool with the aim of achieving universal population coverage by 2030. The proposed benefit package is quite wide and includes basic outpatient and inpatient services and maternity. Outpatient services include consultation fees, laboratory investigation, drug administration and radiological examination, among others. Inpatient services include bed and theatre charges, nursing care, fees for personnel (physicians and surgeons) and drugs, among others. Utilization of these services is expected to increase gradually as more people are covered. There will be no co-payments for using public sector facilities but those who choose to use private sector services are expected to co-pay between 2% of the costs for low-cost private sector facilities to 90% of the cost for high-end private facilities. Although the option of using private providers with co-payments exists, there were proposals within the NHIF to restrict NHIF services to public sector facilities only to address cost-escalation. Should this be the case, there is expected to be pressure to significantly improve the quality of public sector services which currently are perceived to be of poor quality and lack value for money. Scenario 2: Non-contributory system: predominantly tax funded system Under the alternative scenario, government revenue is complemented by the existing statutory premium deductions from formal sector workers and pensioners. Specifically, general government revenues are meant to provide coverage for informal sector workers and the indigent population. Both government revenue and health insurance contributions by formal sector workers will be pooled into the NHIF, which will purchase services for the whole population. The non-contributory scenario is expected to rapidly increase population coverage and utilization of services and should therefore be accompanied with efforts to rapidly improve public sector services. Public spending on health care is also expected to grow rapidly to meet rising demand for health services for the entire population. The benefit package considered is similar to the one in Scenario 1, consisting of essential services but with emphasis on the use of public sector facilities. Such restriction, as with the case with Thailand’s universal coverage scheme [26], is expected to save costs more than it would in a contributory system. The assumption is that there will be no co-payments for those who use public facilities. On the other hand, wealthier individuals are likely to use private services to complement those that they are entitled to from the public sector.	

Expected future changes in disease burden from communicable to non-communicable diseases (NCDs) as well as management of revenue and expenditure were beyond this model. These however, are expected to have some influence on health seeking behavior and health care costs. To make up for these expected changes, the model projections were benchmarked using data from developing countries such as Thailand and Sri Lanka, which have experienced transition from infectious diseases to NCDs and have relatively well managed health systems.

### Data sources

The data sources included national health and economic surveys of various developing countries and data bases of international institutions such as the World Bank, World Health Organization (WHO), Organization for Economic Cooperation and Development (OECD) and the International Monetary Fund (IMF). A number of reports and peer-reviewed journals also provided data for the simulation. Key input data and assumptions were based on various population parameters including population growth rate, various population categories (indigent, formal sector workers and informal sector workers) as well as macroeconomic indicators, healthcare unit costs, health services utilization rates and health insurance coverage (Tables 2 & 3).

**Table 2:** Key input data and assumptions for UHC financing scenarios

Input	Contributory scenario	Non-contributory scenario
Population	1. Baseline total population size is 44.4million [27] and growing at an annual rate of 2.5% [28] to reach 56.8million and 67.6 million in 2023 and 2030 respectively. 2. At baseline, the distribution of the total workforce was as follows: formal sector workers (23.2%), pensioners (5.5%) and informal sector including indigents (71.3%) [29]. The annual growth rate in formal sector employment is estimated at 0.5% on average from the baseline until 2030, although private sector employment would grow at a much faster rate than in the public sector. The informal sector workforce (which also includes indigents) was estimated to decline at an annual rate of 0.37% between 2013 and 2030 based on trends since 2004 [29–31]. Twenty percent of the population is considered indigent at the baseline and this followed a similar declining pattern as the informal sector. The National Bureau of Statistics considers indigents as a part of the informal sector hence the similar growth trajectory. 3. Estimates on growth rates of wages were set at 5% per annum for the formal sector and pensioners at 3% per annum [32, 33].	
Macro-economy	Real GDP growth rate is estimated at an average of 5.0% per annum between 2013 and 2030 and interest rates were set at 5% per annum on average [34].	
Health care unit costs	Unit costs for outpatient and inpatient services for public, non-profit and for-profit facilities were estimated based on evaluation of local studies on unit costs [35]. The unit costs were inflation-adjusted to 2013 prices and projected to estimate prices for 2030. However, a higher growth rate (14.6%) in unit costs was used for public sector services because these are currently under-resourced and significant increase in public funding is required to make quality services available and affordable for all. Average unit costs for private facilities increased at half the rate for public sector (i.e. 7.3%) from 2007 to 2013. The unit costs were as shown in Table 3.	
Utilization rates	Utilization rates were based on analysis of government documents and comparative analysis of the rates in other low- and middle-income countries (LMIC). The rates, on average, were 3.1 outpatient visits per capita per annum at the baseline [6] increasing to 4.0 in 2023 and finally to 4.3 OP visits per capita per annum in 2030. Average annual inpatient days per capita were 0.255 at baseline, 0.287 by 2023 and 0.305 IP days per capita per year by 2030. The utilization rates were projected until 2030 based on current utilization trends in Kenya and triangulated by utilization data from developing countries such as Rwanda [36] and Thailand [37].	
Health insurance	1. Formal sector contributions gradually increased from the current level of 2.4% of gross pay at the baseline [38] to a more realistic contribution rate of 6.5% from 2017 onwards 2. Pensioners contributions were varied from 2.4% of monthly pensions at the baseline to 4% from 2017 onwards 3. Contributions from the informal sector were set at KSh 6000 per household per annum at the baseline (or KSh 1200 per insured adult, child and principal contributor considering that the average household size consists of five people). The KSh 6000 is the new NHIF contribution rate for the informal sector [38]. These are then increased with inflation 4. Annual government subsidies per exempted individual were put at KSh 3000 per annum at baseline. This is based on estimated current government health expenditure per capita. With an average household size of 5, this would be equivalent to KSh15000 per household at the baseline. The subsidy amount was automatically increased in the model in line with inflation. Note: The KSh6000 is the new NHIF contribution amount per household as from April 2015 and already seems like it could be high for many informal sector workers and the KSh15000 is based on current government spending on health, divided by the ‘uninsured’ (informal sector & indigent population). Government contribution per exempted person is higher than contributions from the informal sector because contributions from the latter are regarded as what they can afford and not necessarily what should adequately fund a given package of care. 5. Administrative costs gradually decreased from 12% of total revenue at the baseline to 8% by 2023 and 7% from 2024 onwards. Although the NHIF administrative costs are currently very high (over 35% of total revenue) the study opted for a more realistic figure of 12% at the baseline given that NHIF data validity and reliability is questioned; i.e. large surplus is subsumed under administrative costs [39]. The 12% estimate at the baseline is also within the range of 2–17% of total expenditures on administrative costs in middle-income countries [39]. We use the international best practice to estimate administrative costs for the entire period of the simulation even though the WHO-GTZ mission to Kenya in 2004 recommended administrative costs at 5% of total health insurance expenditure within five years of implementation. The international best practice for administrative costs should range from 10 to 12% of total health insurance expenditure (MOH-PER, 2007) so based on the international best practice it was estimated that administrative costs for the contributory system would be 12% for the first two years of implementing UHC then with efficiency improvements continue to drop off to 10%, 9% (2018–2020) and 8% (2021–2023) and 7% from 2024 to 2030.	1. Formal sector contributions gradually increased from 2.4% of gross pay at the baseline to stand at 6.5% from 2017 onwards 2. Pensioners’ contributions were varied from 2.4% of monthly pensions at the baseline to 4% from 2017 onwards 3. Government subsidies per exempted individual were put at KSh 3000 per annum at the baseline and automatically increased in the model in line with inflation 4. All informal sector workers and indigent populations were exempted 5. Administrative costs reduced from 12% to stay at 5% throughout the simulation consistent with estimates for such costs under non-contributory financing for UHC [39].

The assumptions made under each variable; i.e. population, macro-economy, health care unit costs, utilization rates and health insurance for each scenario, determined the outcome in revenues and expenditures. As such, there was no need for assumptions on revenue and expenditure for each scenario

**Table 3:** Projected unit costs for OP visits and IP days per capita per annum (Constant prices)

Facility	Base year 2013	2023	2030
Dispensary (OP)	394.15	679.67	780.72
Health Centre (OP)	505.14	871.07	1000.58
County Hospital (OP)	1078.24	1859.32	2135.77
County Hospital (IP)	4610.83	7950.93	9133.12
National Hospital (OP)	3191.68	5503.74	6322.07
National Hospital (IP)	11147.08	19222.07	22080.12
Private non-profit (OP)	1205.66	1469.69	1575.71
Private non-profit hospital (IP)	6058.83	7385.68	7918.45
Private for-profit clinic (OP)	1297.23	1581.32	1695.39
Private for-profit clinic (IP)	18116.97	22084.49	23677.56
Private for-profit hospital (OP)	2952.34	3598.89	3858.50
Private for-profit hospital (IP)	20606.13	25118.76	26930.71

More than simply inflation-rating unit costs from 2007 to 2030, we used a Ministry of Health task force in 2012 report that estimated that public facilities should get about twice the funding that they currently receive to improve service delivery to acceptable levels [10]. Such rapid investment in the health sector may not be possible given past trends in funding from the government. On this basis we can assume that, while funding for the health sector may not more than double as recommended by the taskforce, the funding would increase at a rate twice higher than the prevailing inflation rates to better approximate future unit costs and the need to anchor universal coverage. On this basis a 14.6% rate from 2007 to 2013 was used to estimate public provider unit costs for base year 2013. Following a linear projection, implementation of strategic purchasing as more people get covered and coupled with competition from improved services in the public sector will put pressure on the private sector to have reasonable prices such that, over a period of time, there would be a tendency towards convergence of unit costs between public and private services. On this basis it was assumed that average unit costs for private facilities increased at half the rate for public sector (i.e. 7.3%) from 2007 to 2013.

### Limitations of SimIns

A notable limitation with SimIns is that it only models for 10 years and although the modelling work extended to 2030, there are problems in transition between the two models especially with regard to financial estimates. This was resolved by using a Microsoft Excel sheet to calculate revenue and expenditure estimates based on trends from 2013 to 2023. The other notable weakness mainly with SimIns Basic is that it does not provide a complete picture of the total health expenditure as it does not include private health expenditure outside of health facilities (e.g. on self-treatment).

### Sensitivity analysis

Sensitivity analysis was conducted by adjusting contribution and government subsidy rates to address potential deficits in both scenarios (Table 4).

**Table 4:** Key variables for sensitivity analysis to ensure sustainability

Scenario	Strategy for sustainability
Contributory scenario	1) Formal sector contributions increased from 2.4% at the baseline to 6% then 7.5%, 8% and finally 11% of gross pay from 2019 onwards. 2) Pensioners’ contributions were varied from 2.4 to 4% and finally 5% of pensions from 2019 onwards. 3) Annual subsidies were put at KSh 4500 per exempted person at the baseline and reached KSh 15000 per exempted person within a decade. 4) Annual contributions from the informal sector were started at KSh 2000 at the baseline and increased to KSh 3401 in 2023 and KSh 4536 in 2030 per insured adult, child and principal contributor. 5) Administrative costs were varied from 12% of total revenue at the baseline to 11, 10 and 8% by 2023 and 7% from 2024 onwards.
Non-contributory scenario	1) Same as 1–3 above. 2) All informal sector workers and indigent populations were exempted. Cost per exempted is the same for all scenarios but the difference is that in the contributory scenario has fewer exemptions (indigents only) while the contributory scenario has both informal sector and indigents exempted. This has important implications on amounts of revenues generated. 3) Administrative costs reduced from 12% to stay at 5% throughout the simulation.

## Results

### Overview of population coverage and service utilization

Population coverage and service utilization are important influencers of costs. The modelling divided population coverage into three different population groups: formal sector workers, informal sector including the indigent population, and pensioners. In both scenarios, coverage for formal sector workers and pensioners follows a similar pattern because it is more straightforward to enforce membership through payroll deductions. It was assumed that with strict laws on coverage and increased demand for health insurance, every employer will be compelled to comply with government regulations to declare 100% of their workers. On the other hand, the coverage trends for informal sector populations take different trajectories under each scenario. Under the non-contributory scenario, coverage of informal sector and indigent populations is expected to increase to 98% in the second year of implementation because it targets everyone and is easier to implement compared to the contributory scenario where coverage increases gradually throughout the simulation (2013–2030). The assumption underlying this level of coverage is based on recent universal services in Kenya including free maternity care and free primary care. The latter has benefited all Kenyans within the first year of implementation. Under the contributory scenario, population coverage increases from 19.5% at the baseline to 68.5% in the 10th year of implementation. About 98% population coverage (universal coverage) is achieved in the 17^th^ year of implementation of the contributory system.

With regard to utilization of services, from a baseline of 3.1 OP visits per capita per year, the total utilization of OP services across the population, on average, are projected at about 4.00 annual visits per capita by 2023 and 4.31 annual visits per capita at the end of the simulation. Inpatient days per capita per year at the baseline were set at 0.25 as per the most recent government survey [6] and projected to increase to 0.27 in 2023 and 0.29 in 2030 (Table 5).

**Table 5:** Annual utilization of outpatient and inpatient services (2013–2030)

	Baseline	2023	2030
Outpatient visits per capita	3.10	4.00	4.31
Insured	3.26	4.21	4.54
Uninsured	2.93	3.78	4.07
Inpatient day per capita	0.25	0.27	0.29
Insured	0.33	0.35	0.38
Uninsured	0.17	0.18	0.20

Based on the benchmarks in Table 5, the modelling result shows marked differences between contributory and non-contributory scenarios especially in the total number of inpatient days. At the baseline, both scenarios have 132.9 million annual OP visits, which in the 10th year (2023) increase to 231.7 million and 239.1 million visits for contributory and non-contributory scenarios, respectively. This difference in numbers between the two scenarios is due to the fact that the non-contributory scenario reached universal status (97% coverage) as opposed to the contributory scenario where only 54% of the population is covered by 2023. However, the differences in OP visits between the two scenarios is minimal for the insured (4.20 visits per capita per annum) and the uninsured (3.80 per capita per annum) in 2023. The differences are much greater for inpatient services (0.35 inpatient days per capita per year for insured versus 0.18 for uninsured).

### Health insurance revenue and expenditure (Constant prices)

**Fig. 1:**
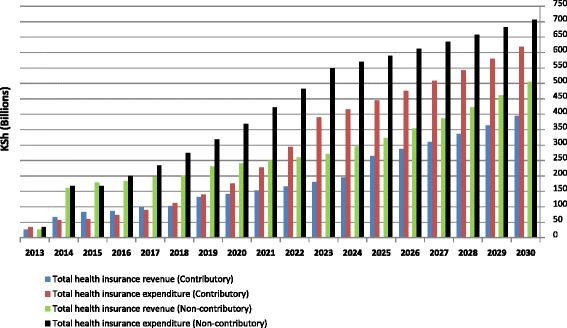
Health insurance revenue and expenditure

Utilization rates and unit costs are major drivers of expenditure. The more people that are covered the more money is required to meet the expenditure. In total, barring any cost-containment measures, Fig. 1 indicates that the contributory scenario in 2030 would generate KSh 395 billion in revenues compared to about KSh 619 billion in expenditure. The non-contributory scenario, on the other hand, would have about KSh 505 billion in revenues compared to about KSh 706 billion in expenditure.

There is higher expenditure over a period of time under the non-contributory scenario than there is in the contributory scenario because of the higher number of people covered in the former scenario. The gap in expenditure between the two scenarios is however, narrowed at the end of the simulation because both have achieved near-universal population coverage and costs and utilization levels are the same. Noting that the non-contributory scenario was financially unsustainable (had higher expenditure than revenue) from the beginning as opposed to the contributory scenario which lasted a few years before running into deficits (Fig. 1), it implies that government subsidies for the informal sector and indigent populations as modelled are insufficient and would have to be increased for revenue to match or exceed expenditure.

**Fig. 2:**
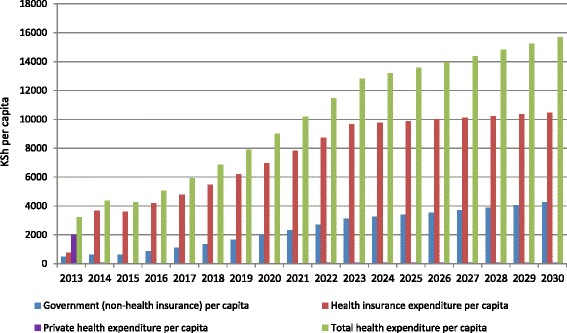
Health expenditure patterns: projections for non-contributory scenario. [*Government non-health insurance expenditure: Costs such as reserves and administrative costs, among others. They are not directly used in the production of health*]

**Fig. 3:**
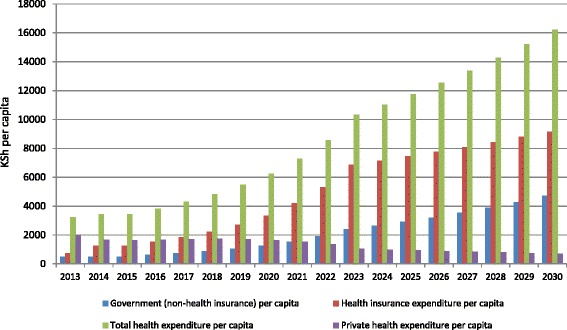
Health expenditure patterns: projections for contributory scenario

Analysis of expenditures per capita (Figs. 2 and 3) suggest that a non-contributory scenario would cost less overall with total health expenditure (THE) per capita at health facilities coming to about KSh 15,700 (US$ 157) at the end of the simulation compared to about KSh 16,200 (US$ 162) for the contributory scenario. Although the differences in THE per capita are not statistically significant (*P* = 0.323), there are important budgetary implications with any slight cost variations over the long term. Private health expenditure per capita reduces rapidly in the non-contributory scenario from KSh 1985 at the baseline to KSh 59 by 2030. On the other hand, private spending in the contributory scenario steadily reduces from KSh 1985 per capita at the baseline to KSh 703 per capita at the end of the simulation. Government (non-health insurance) expenditure per capita is higher in the contributory than in the non-contributory scenario, which partly contributes to the overall higher THE in the contributory scenario. It indicates that the government would have to spend more money in non-health insurance areas to support a contributory scenario. However, overall the non-contributory scenario has higher total government subsidies for health insurance because of the large population that is exempted from contributions and because government subsidies would need to be at a rate higher than the informal sector would have been able to personally contribute in premiums under the contributory scenario.

### Sensitivity analysis: strategies to sustainably finance UHC for each scenario

The two scenarios have large deficits a few years into implementation and are therefore unsustainable under prevailing levels of contributions and subsidies. Both scenarios can be sustainable but there is a limit to which contribution rates or taxes can be increased to ensure that revenues match or exceed expenditure. Therefore, the scenario that breaks even at the lowest cost is the most viable option for UHC. The study explored the option of increasing both premium contributions and government subsidies to ensure that revenues match expenditure at the lowest cost. The rationale for all the adjustments was to ensure that expenditures are not more than revenues for either scenario. The deficits to be addressed were not categorized and stepwise adjustments were done. The scenario that broke even at the lowest cost was considered the least expensive even though other factors not considered in this study could be at play. Annual subsidies were put at KSh 4500 per exempted person at the baseline and reached KSh 15000 per exempted person within a decade. Because there were more exempted people under the non-contributory scenario than were in the contributory scenario, the revenue potential for the non-contributory scenario is higher than for the contributory scenario.

**Fig. 4:**
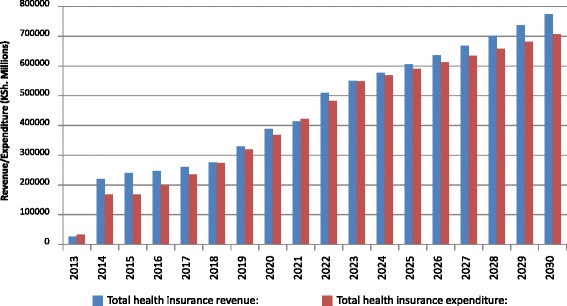
Sustainable financing: non-contributory scenario

With adjusted levels of contributions and subsidies, a non-contributory scenario shows considerable sustainability throughout the simulation with total revenue exceeding total expenditure by about KSh 67 billion in 2030 (Fig. 4).

**Fig. 5:**
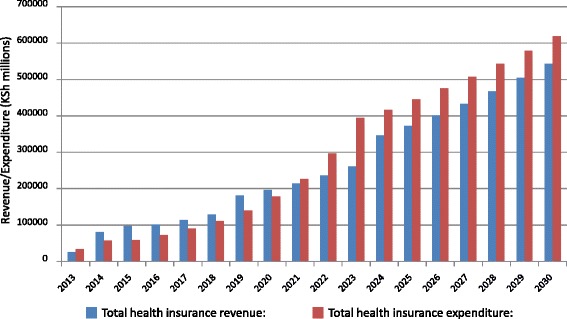
Sustainable financing: contributory scenario

The main reason for sustainability in a non-contributory mechanism is the large amounts of government subsidies paid into the pooling and purchasing institution on behalf of informal sector and indigent populations. However, total government subsidies are only 58% of the total health insurance revenue with total contributions from formal sector workers and pensioners accounting for about 41% of the total health insurance revenue. The contributory scenario, on the other hand, even with increased contribution rates is unsustainable after 2021 with total expenditure exceeding total revenues by about KSh 74.8 billion by 2030 (Fig. 5).

The reason for the differences in revenue and expenditure is that whereas contributions from the formal and informal sector workers are high (91% of total revenues), government subsidies are very low at only 8.20% of total revenue.

## Discussion

The goal of UHC has relevance to all countries. All countries want to reduce the gap between need and utilization of health services, improve financial protection and quality of services for all citizens [11]. In reforming health systems to address these key issues, countries should give particular attention to the financing functions, to ensure that the most efficient, equitable and sustainable means of financing the health system are adopted.

The results indicate that the non-contributory scenario has the potential to generate higher total revenues throughout the simulation. This is because contributions from the informal sector under the contributory scenario are very limited (at KSh6000 per household at baseline) compared to what the government pays in subsidies on behalf of this population group (KSh15000 at baseline) under the non-contributory scenario. This implies that in circumstances where informal sector workers would be required to contribute, the revenue generated would be minimal as contributions are not based on amounts that can finance a given package of care but rather on the economic circumstances in the informal sector. Second, as noticeable in the second year of implementing the non-contributory scenario, revenues dramatically increased because the government puts in more money in subsidies for informal sector and indigent populations as opposed to the gradual increments in contributions from informal sector workers in the contributory scenario.

In theory, both models (contributory and non-contributory) can provide sustainable health financing options for UHC. In practice, the contributory scenario in particular, poses serious challenges in contexts with a large informal sector. The results presented in this paper suggest that the contributory scenario would be (i) more costly than the non-contributory scenario for same level of utilization among the insured); (ii) much slower than the non-contributory scenario in advancing population coverage; (iii) contribute to inequities in financing and utilization. The inequities in utilization would persist until the entire population is covered but in the meantime, those already covered would be enjoying higher utilization rates than those not covered. In terms of financing, inequity is unavoidable especially in the informal sector where, in absolute figures, the contributions are flat-rated, which constitute regressive financing. Previous studies indicate that implementation of a contributory financing mechanism in countries with large informal sector is costly and problematic if not outright impossible because of the difficulties associated with identifying those who should contribute and collecting premiums in the sector [7, 12].

Even if the informal sector were organized to make revenue collection possible under a contributory mechanism, the results indicate that for revenues and expenditure to break even, the premium contribution rates for both formal and informal sector populations would have to drastically increase. Increases in contribution rates are likely to further complicate efforts to implement a contributory financing mechanism, and may exclude the poor, in a context where mechanisms to target the poor are not well developed. Increasing premium rates, for example from 2.4 to 11% of gross salaries, is likely to face resistance from the public because there is already extensive resistance to the prevailing NHIF rates both from employers and contributors [13]. It is also noteworthy that any increase in contribution rates should be carefully assessed in terms of the burden it will place on contributors. However, the contribution rates should be realistic in terms of financing a given package of services. Granted, an affordable benefit package should be defined, but the package needs to be comprehensive enough to protect service users from financial risks. The reality in the Kenyan context is that the current premium rates under the NHIF are quite low averaging 2.4% of gross pay for formal sector workers yet the revised benefit package is very generous, which raises concerns about the sustainability of the NHIF. As Kenya embarks on the UHC journey, it is important that the benefit package offered to the population can be financed adequately from the total available resources.

Less government subsidies and more contributions from the informal sector in the contributory model is counterintuitive because the flat-rate premiums are unaffordable to most low-income groups. Although data from OECD countries indicate that government subsidies may be as low as 5.0% of total health expenditure in contributory systems [14], the Kenyan case is different because the majority of potential contributors are in the informal sector where incomes are not only low but difficult to target in a contributory health financing system. There is a limit to which premium rates in the contributory scenario can be increased to ensure sustainability. At 2.4% of gross pay, there were serious resistance to new NHIF contributions [13]; at 11% of gross pay as suggested by the model, the contributory system still cannot raise revenues that match expenditure. Such an increase is also likely to face very stiff resistance from the population and unlikely to be implemented. Therefore, under the contributory financing approach, for revenue and expenditure to break even, the government needs to significantly increase its subsidies both for the indigent and for those who could make some contribution but only a small amount. In essence, increasing premium contribution rates to a level that revenues match expenditure would be counterproductive; would be unaffordable and likely to cause resistance from the entire population and each time will have to be strongly justified to be effected if at all.

Given the aforementioned challenges with a contributory approach to UHC, the Kenyan government should consider the non-contributory approach as an alternative option for moving towards UHC. For historical reasons and the perception that tax revenues alone cannot finance UHC, many governments in developing countries are in favor of contributory schemes [4, 5, 8, 15]. However, recent evidence [16, 17] suggests that the non-contributory approach could be the best option to make progress toward UHC in many developing countries that are characterized by large informal sector populations. Moreover, a centrally managed non-contributory health financing system has considerable potential to promote equity across geographic regions and socioeconomic groups [18]. The modelling results indicate very rapid population coverage, which is indicative of achievement of equity in service coverage for the large majority of the population over a short period of time. Although the pace of extending population coverage in the non-contributory model is overly ambitious, coverage extension would be far slower in a contributory approach in the Kenyan context. Besides, most revenue would be from direct and indirect taxes, both of which are generally progressive especially in low-income countries [19].

The key issue for Kenya is whether there is political will to pursue this option, because no matter how evidence-based and technically sound the proposal for a non-contributory financing approach is, successful implementation will be dictated by the political context [11] and the priority given to the health sector. First, the past and current trends in health sector funding suggest that the health sector receives less priority in budget allocation compared to other social sectors. Unless there is government commitment to UHC through the non-contributory approach it is unlikely that Kenya will achieve UHC by 2030 as projected by the government. Second, with the recent devolution, county governments not only have semi-autonomous governance systems but also independent control of respective budgets and have expressed the desire to run their health programs according to their own designs. While devolution creates opportunities to take services to the people and potentially improve efficiency and accountability, it poses challenges for pooling. Thus the design of health financing reforms in Kenya should carefully consider the political context and the governance structures to minimize fragmentation and support financial risk protection and access to needed, quality health care for all Kenyans.

### Recommendations for additional revenue to finance UHC under non-contributory mechanism

The non-contributory financing approach seems to be the most viable option for Kenya under the prevailing circumstances. This approach requires very heavy financial commitments and it is important to consider where such large funding is likely to come from to allow for large government subsidies to the health sector. A number of sources could be assessed as potential avenues to increase fiscal space for health care in Kenya including shifting expenditure from other government functions to the health sector, increasing health insurance premium rates as well as tax rates, exploring a number of innovative financing strategies, and improving efficiency in revenue collection and use of available resources.

Increasing general tax and dedicated tax rates may look unavoidable under the circumstances of expenditure outstripping revenue to make financing unsustainable. However, the current pattern of government spending suggests that before considering increased taxes or premium rates there is the possibility of increased government funding for the health sector under prevailing budgetary conditions mainly by shifting funding from other government sectors to the health sector. Expenditure by functions of government suggest that Kenya devotes large proportions of the budget on functions other than health care (general public services (17%), economic affairs (22%), defense (5.3%) and public order and safety (7.6%) [20]). The EU comparably spends lower on these functions with a large budget for health care (general public services (14%), economic affairs (8.8%), defense (2.9%) and public order and safety (3.8%) [21]). The expenditure by function strongly indicates that Kenya can possibly re-evaluate its expenditure on various government functions with the view to giving greater priority than at present to the health sector along the lines of developing countries with UHC systems. A comparative analysis with developing countries such as Ghana, Rwanda, Costa Rica, Brazil and Thailand, which have all taken steps to move towards UHC, indicates that Kenya ranks worst in terms of key health expenditure indicators. Noting that total government expenditure in Kenya accounts for 31% of its GDP, well above those of countries such as Costa Rica and Thailand which have universal health systems [22, 23], it should be a question of more priority to the health sector rather than taking the first and more difficult option of increasing tax rates.

Innovations in financing including non-tax sources such natural resources, levies on mobile phone companies, tax on international money transfers and surcharge on VAT, among others, are potential additional sources of revenue for the health sector [2, 24]. The latter is often considered in countries with large informal sectors given that all residents pay some VAT.

Further commitment to financing UHC would be demonstrated if the government could honor the Abuja obligation of allocating at least 15% of the national budget to the health sector. The Abuja target is not a guarantee for adequate financing and service delivery and should be interpreted within specific contexts. Alternatively, the government could spend at least 5% of GDP on healthcare from domestic resources. The 5% of GDP is the financial estimate required to adequately finance UHC [16]. A Ministry of Health (MOH) Taskforce [25] estimated that KSh 217 billion (about US$ 2.17 billion) would be adequate to provide necessary health services to all Kenyans as of 2013. Based on the analysis of Kenya’s GDP and national budgets from 2013 to 2016 and going by the taskforce recommendation, spending the equivalent of 5% of GDP or 15% of the national budget would mobilize more than adequate resources to fund UHC in Kenya. The results of the simulation under the non-contributory scenario indicates that the amounts required to finance UHC in 2014, 2015 and 2016 would be KSh 220.8, 239.6 and 246.6 billion, respectively. These amounts are closer to the 5% of the GDP or 15% of the budget and slightly higher than the MOH estimates. These are tangible options for raising adequate revenue for UHC in Kenya and should be aggressively pursued by advocacy groups, policy makers and the political leadership.

Future research needs could focus on ways to improve efficiency in revenue collection especially under the context of devolution as well as the analysis of fiscal space with special attention to the health sector. Further research could focus on modeling current health financing policy for UHC.

## Conclusion

There is no doubt that to raise adequate revenues to finance UHC, the health sector requires substantial financial input to be able to provide access to quality health services with financial protection for all. It is noteworthy that any mandatory prepayment in this context– whether a premium to the NHIF or taxes to the general government basket– are regarded as ‘government funding’ (e.g. both are included in government expenditure on health measures in national health accounts). So whether contributory or non-contributory, increased mandatory prepayment funding is required. In this context, efforts towards increasing fiscal space for health are required. In Kenya, the government is in favor of a contributory financing system for UHC. However, given the structure of the labor force such an approach may not be feasible. Furthermore, under the proposed contributory arrangement, there is considerable silence over how to effectively identify groups falling below a certain income threshold for subsidies and whether there will be steps to formalize labor. In many other contexts, identifying the poor for subsidies has proven difficult in and is unlikely to be successful in Kenya.

The benefits of adopting a non-contributory financing approach, although more expensive at the beginning than the contributory mechanism, are clear in the long-term. The non-contributory mechanism comes at a lower overall cost than a contributory approach. The associated high start-up costs may be addressed through shifting expenditure from other sectors of the government, innovative financing, improving efficiency in spending including curbing corruption and other leakages as well as initiating phased coverage of specific services. It is noteworthy that there are human resources for health (HRH) deficits in Kenya and while facility capacity might meet the health needs of a large percentage of the population covered under a non-contributory scenario in 2 years, the HRH deficits will be much tougher to resolve in 12-24 months.

## Abbreviations

NHIF: National Hospital Insurance Fund
SIMINS: Simulation insurance
UHC: Universal health coverage
WHO/GTZ: World Health Organization/German Technical Agency
